# Interposition Patches for Massive Rotator Cuff Tears: Helpful or Not? A Prospective Study of 164 Interposition Polytetrafluoroethylene Patches

**DOI:** 10.1177/23259671251333801

**Published:** 2025-06-20

**Authors:** James Bilbrough, Ala Hawa, Mina Shenouda, Christyon Hayek, George A.C. Murrell

**Affiliations:** * Orthopaedic Research Institute, St. George Hospital Campus, University of New South Wales, Sydney, Australia; † Orthopedics Division, Department of Surgery, Faculty of Medicine, Yarmouk University, Irbid, Jordan; Investigation performed at the Orthopaedic Research Institute, St. George Hospital, *University of New South Wales, Sydney, Australia*

**Keywords:** interposition patch, irreparable rotator cuff tear, massive rotator cuff tear, polytetrafluoroethylene patch

## Abstract

**Background:**

It is undetermined how well interposition polytetrafluoroethylene (PTFE) patch repairs for massive and irreparable rotator cuff tears perform in the longer term and whether the procedure can prevent proximal humeral migration, glenohumeral arthritis, and conversion to reverse total shoulder arthroplasty.

**Purpose:**

To answer the following questions: (1) Do interposition PTFE patch repairs inserted for massive and irreparable rotator cuff tears improve patient pain and function, shoulder strength, and range of motion? (2) How long do interposition PTFE patches last? (3) Do interposition PTFE patches prevent proximal humeral migration and glenohumeral arthritis? (4) Do interposition PTFE patches prevent patient conversion to reverse total shoulder arthroplasty?

**Study Design:**

Case series; Level of evidence, 4.

**Methods:**

We conducted a prospective cohort study of 164 consecutive patients with interposition PTFE patch repairs for massive and irreparable rotator cuff tears, with a median follow-up time of 26 months. Patient-reported pain and function, shoulder strength, and range of motion were assessed preoperatively, at 6 months postoperatively, and at the final follow-up. Ultrasound was used to assess PTFE patch integrity at 6 months postoperatively and at the final follow-up, and shoulder radiographs were taken at 6 months postoperatively and at the final follow-up. Kaplan-Meier survival analysis was used.

**Results:**

Patients with intact PTFE patches on ultrasound had greater improvements in pain and function, strength, and range of motion at the final follow-up (median, 26 months; range, 6 months to 19 years) when compared with patients with nonintact PTFE patches (*P* < .05). Of 164 interposition PTFE patches, 50 (30%) functionally failed at a median time of 5 years. In the whole cohort, Kaplan-Meier analysis estimated that the median survivorship time of these PTFE patches was 7.4 years. Furthermore, patients with intact interposition PTFE patches demonstrated a 21% lower severity of glenohumeral arthritis (*P* = .03) and a 46% lower incidence of proximal humeral migration (*P* < .001) than patients with nonintact interposition PTFE patches. At the final follow-up, 93% of participants were free from conversion to reverse total shoulder arthroplasty after interposition PTFE patch repair for a massive and irreparable rotator cuff tear.

**Conclusion:**

Based on the results of this study, interposition PTFE patch repairs for massive and irreparable rotator cuff tears were efficacious at improving patients’ pain and function, strength, and range of motion. Patients with intact interposition PTFE patches were associated with reduced severity of glenohumeral arthritis and reduced incidence of proximal humeral migration. At the final follow-up, 93% of participants were free from conversion to reverse total shoulder arthroplasty.

Massive rotator cuff tears are debilitating, often presenting with significant loss of shoulder strength and function.^6^ These tears often result in proximal humeral migration and glenohumeral arthritis, for which the current management is reverse total shoulder arthroplasty.^3,17^ Interposition patches are a potential solution for managing massive and irreparable rotator cuff tears, which aim to bridge the defect between the rotator cuff tendon and footprint on the greater tuberosity. Polytetrafluoroethylene (PTFE) patches are synthetic grafts with low rates of infection, strong mechanical properties, and greater construct integrity than biological and synthetic alternatives.^1,7,8,11,14^ To date, few studies have determined how well interposition PTFE patches perform in the longer term with a large sample size and whether they prevent patients from conversion to reverse total shoulder arthroplasty.

This study aimed to answer the following questions: (1) Do interposition PTFE patches inserted for massive and irreparable rotator cuff tears improve patient pain and function, shoulder strength, and range of motion? (2) How long do interposition PTFE patches last? (3) Do interposition PTFE patches prevent proximal humeral migration and glenohumeral arthritis? (4) Do interposition PTFE patches prevent patient conversion to reverse total shoulder arthroplasty?

## Methods

### Study Design

This was a single-center cohort study that aimed to analyze the outcomes of interposition PTFE patch repairs for massive and irreparable rotator cuff tears. Data was prospectively collected and analyzed retrospectively. All participants provided consent, and ethics was approved (2019/ETH14049).

### Inclusion and Exclusion Criteria

Patients who received an interposition PTFE patch for a massive and irreparable rotator cuff tear by a single surgeon (G.A.C.M.) were included in the study. The principal intraoperative criteria were rotator cuff tears, which were irreparable due to significant retraction and could not be reattached to their footprint with standard rotator cuff repair. The minimum follow-up period was 6 months.

### Cohort

**Figure 1.**
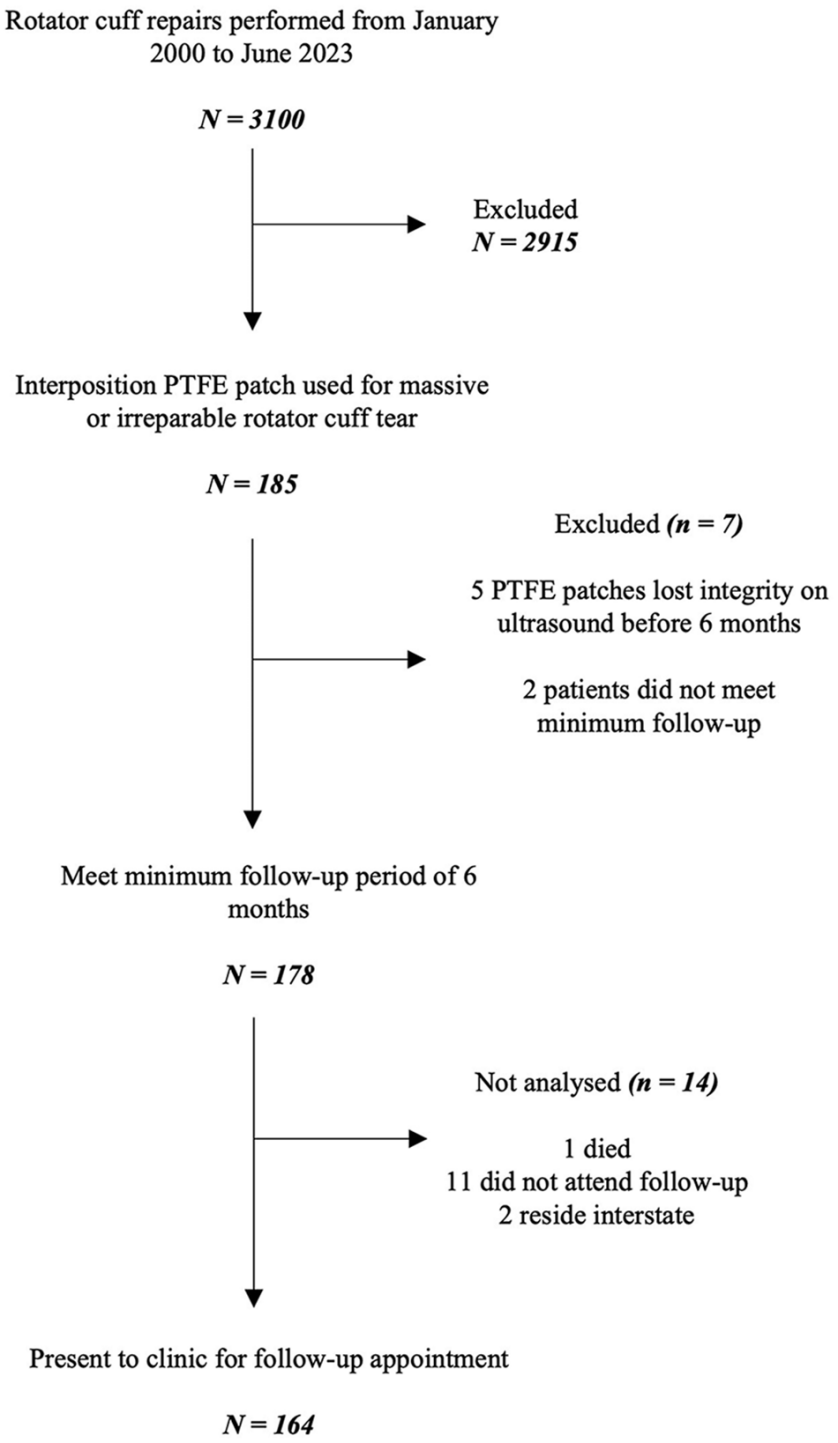
A flow diagram of patient recruitment. PTFE, polytetrafluoroethylene.

**Figure 2.**
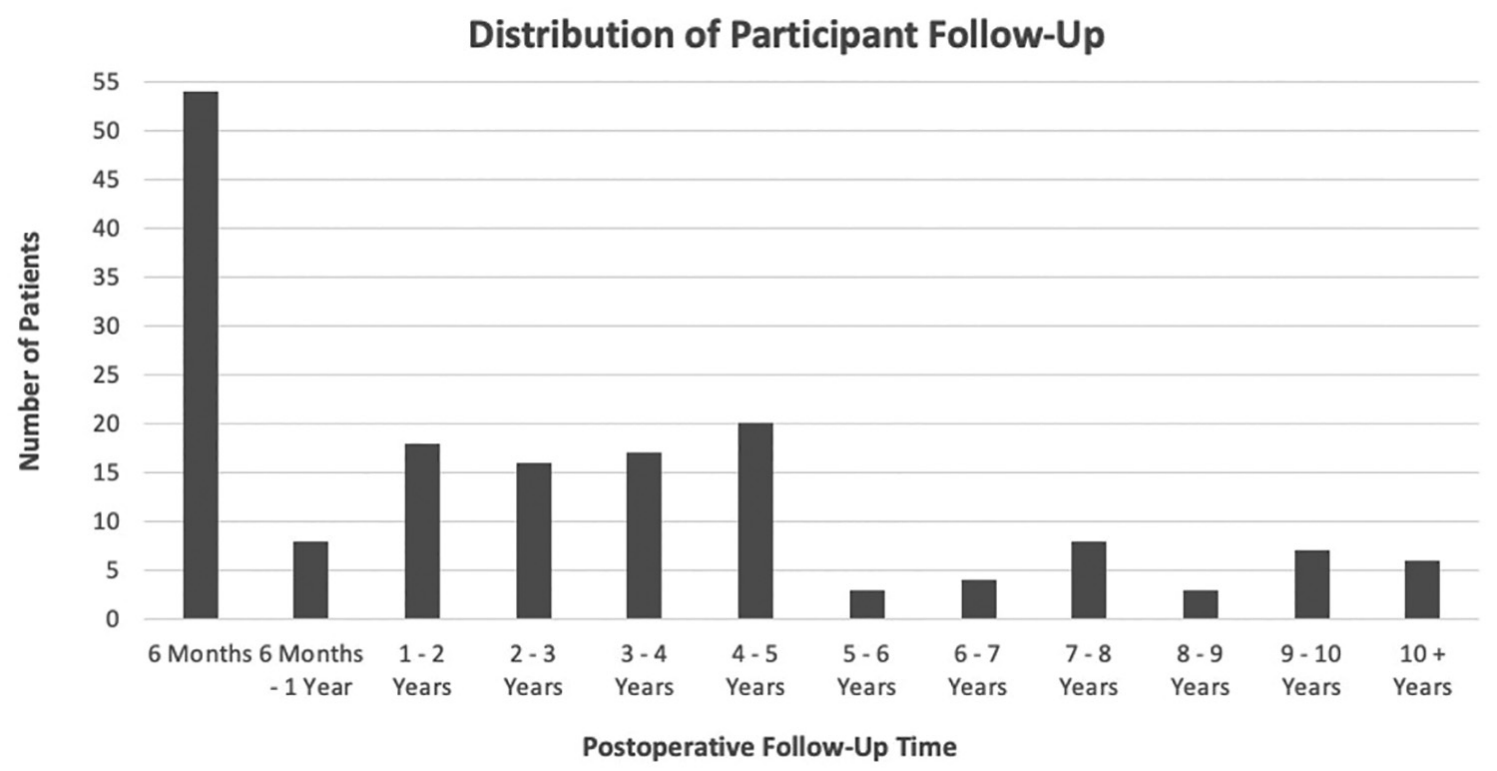
Distribution of the final follow-up for patients with interposition PTFE patch repairs. n = 164; PTFE, polytetrafluoroethylene.

At our institution, 3100 rotator cuff repairs were performed between January 2000 and June 2023 by a single surgeon (G.A.C.M.). Of these repairs, 185 (6%) used an interposition PTFE patch to bridge a massive and irreparable rotator cuff tear. Two patients did not meet the minimum follow-up period of 6 months, and 5 patients’ PTFE patches lost integrity before this time. These 5 patients with early patch failure were included in the final Kaplan-Meier analyses. Thirteen patients were lost to follow-up, and 1 patient died. As such, this study had an overall follow-up rate of 92% and included 164 patients with interposition PTFE patch repairs (Figure 1). The median follow-up time was 26 months (range, 6 months to 19 years) (Figure 2).

### Surgical Technique

**Figure 3.**
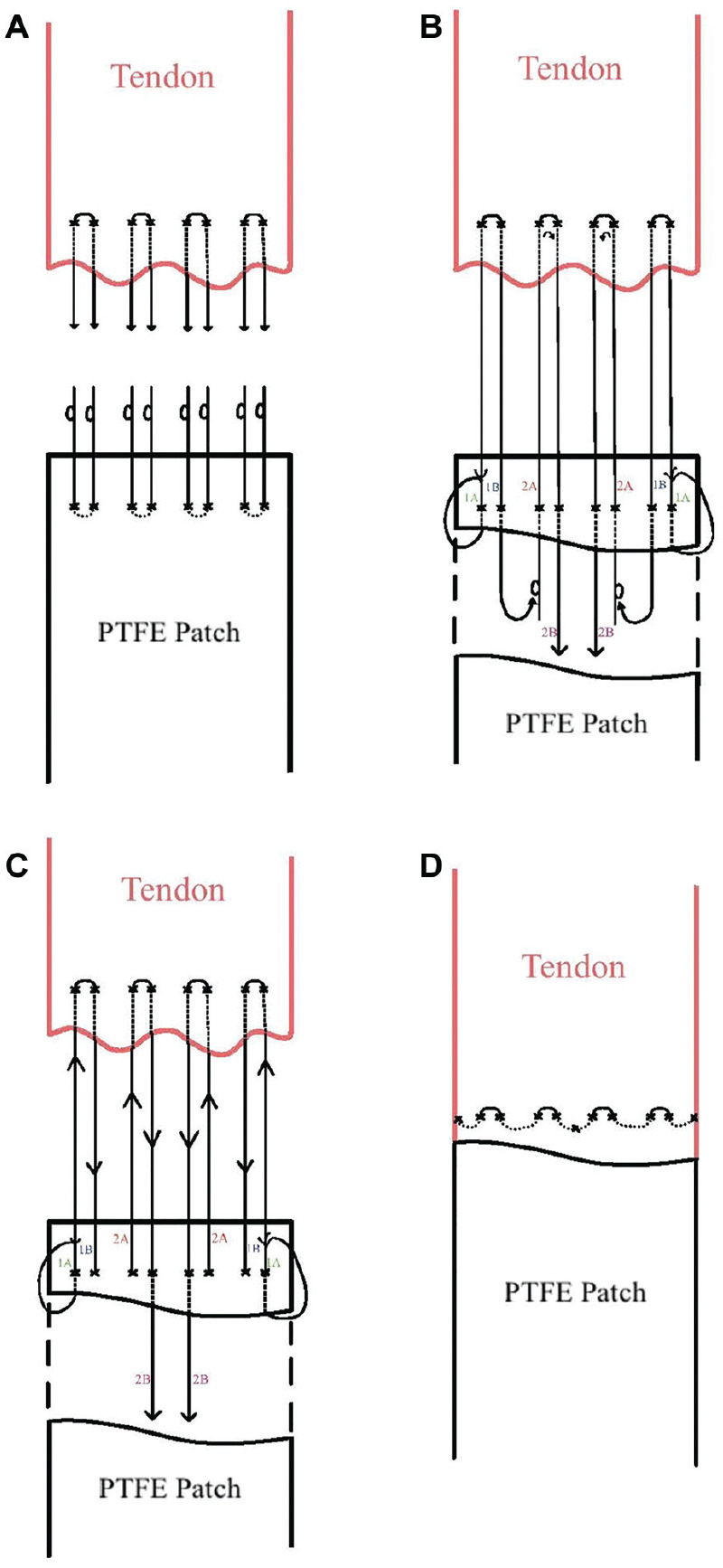
Illustration of the weave repair technique for interposition PTFE patch repair. Free ends of sutures labeled 1A to 2B. (A) Smith & Nephew (S & N) SmartStitch suturing device and S & N Opus SmartStitch M-Connector were used to pass 4 sutures through the tendon edge arthroscopically, ensuring equidistant intervals. A color-alternating pattern (blue-white-blue-white) was used from anterior to posterior to allow for suture orientation. Externally, the same devices were used to pass 4 different sutures through the PTFE patch, using the same color-alternating technique. Suture loops are made at the distal ends of PTFE patch sutures, through which corresponding tendon sutures are shuttled. (B) The outer 2 suture tails (1A) were tied off using a standard square knot; 2 overhand half-hitches followed by 3 underhand half-hitches. The suture tails adjacent to these (1B) were then advanced through the suture limbs medial to them (2A). (C) The patch was then advanced through the arthroscopic portal while pulling on the 2 central suture tails (2B) to ensure a flush position on the tendon. (D) The median suture tails (2B) were tied using a standard arthroscopic square knot. PTFE, polytetrafluoroethylene.

All surgeries were performed as day-cases with an interscalene nerve block and light sedation. The senior author (G.A.C.M.) initially began inserting interposition PTFE patches for massive and irreparable rotator cuff tears by open surgery.^20^ This open method was later replaced briefly by the arthroscopic mattress technique and now, the most frequently used, weave technique (Figure 3). This change in suture configuration was driven by an aim to reduce the number of knots tied arthroscopically, with support from previous studies.^18,20,23^ Standard rotator cuff repair rehabilitation was used for all patients.^5^ In brief, patients were discharged on the same day of surgery in a sling with a small abduction pillow (Ultrasling; DJO). The sling was to be worn full-time for 6 weeks, during which patients were to complete passive range of motion exercises. Loading was initiated at 3 months. No restrictions were imposed at 6 months.

### Outcome Variables

#### Patient-Reported Pain and Function

A standardized questionnaire adapted from the previously validated L’Inslata Shoulder Rating Questionnaire^13^ (Appendix Figure A1) was completed routinely by patients preoperatively, postoperatively, at 6 months, and at the final follow-up.^20^

#### Range of Motion and Strength

Passive shoulder range of motion—including forward flexion, abduction, and internal rotation—was measured visually by trained examiners. Forward flexion and abduction were measured in degrees, and internal rotation was measured by vertebral levels. Strength was measured with a hand-held dynamometer (HFG-45 Force Gauge) (reported in N) at each visit.^16,20^ Supraspinatus strength was measured with the patient's arm at 90° of abduction and 30° anterior to the coronal plane, while internal and external rotation strength were measured at 90° of elbow flexion and the forearm in neutral position.

#### Patch Integrity on Ultrasound

Interposition PTFE patch integrity was assessed at 6 months and at the final follow-up. An experienced musculoskeletal sonographer (M.S.) used a Siemens Acuson S300 ultrasound system coupled with a 12-MHz linear transducer (Siemens Medical Solutions; Mountain View) to visualize the interposition PTFE patch repair using a standard protocol.^2,4^ The patch-to-bone and patch-to-tendon interfaces were classified as “intact” or “nonintact.” The patch was intact if there was no visible defect at either attachment sites. If either site was nonintact or was unable to be identified on ultrasound, the PTFE patch was considered to be nonintact.

#### Functional Failure

An interposition PTFE patch was considered to have functionally failed if (1) the attachment at either the rotator cuff tendon or greater tuberosity had lost integrity on ultrasound or (2) the patient underwent removal of the interposition PTFE patch with or without conversion to reverse total shoulder arthroplasty.

#### Arthritis and Proximal Humeral Migration

True anterior-posterior shoulder radiographs were taken on participants using the Carestream Odyssey High Frequency X-Ray Machine (New York) as described by van de Sande and Rozing.^24^ At the time of interposition PTFE patch repair, glenohumeral arthritis was assessed arthroscopically and graded according to the Outerbridge grading system.^22^ At the final follow-up, arthritis was graded using the Kellgren-Lawrence System.^10,19^ A blinded shoulder surgeon (A.H.) graded the participants’ arthritis and scored the radiographs as “evident” or “not evident” for proximal humeral migration as per Keener et al.^9^

### Statistical Analysis

Participants were divided into “intact” and “nonintact” groups depending on the integrity of their interposition PTFE patch on ultrasound at the final follow-up. Kaplan-Meier analysis was used to analyze interposition PTFE patch integrity on ultrasound, conversion to reverse total shoulder arthroplasty, and functional survivorship of interposition PTFE patch repairs. Patient-reported data, range of motion, and strength were compared with the Wilcoxon signed-rank and Mann-Whitney *U* tests. The Mann-Whitney *U* test was used to compare glenohumeral arthritis, and the chi-square test was used to compare proximal humeral migration and analyze whether PTFE patch functional failure was different between PTFE patch types. Significance was set at *P* < .05. Statistical analysis was conducted on SPSS Statistics for Windows Version 27 (IBM) and GraphPad Prism for MacOS Version 7 (GraphPad Software Inc).

## Results

### Patient Characteristics

Of the 164 participants, 108 (66%) were men and 56 (34%) were women. The mean age at surgery was 64 ± 10 years (range, 39-91 years). There were 98 (60%) right shoulders and 66 (40%) left shoulders. A total of 98 (60%) operations were primary, and 66 (40%) were revision cases. Three of the revision cases were interposition PTFE patch revisions, while 63 were revisions of rotator cuff repairs. All patients had full-thickness tears. The mean tear sizes were 36 mm (SD, 11 mm; range, 10-80 mm) for anteroposterior width and 31mm (SD, 11 mm; range, 10-70 mm) for mediolateral length. The mean tear area size was 12 cm^2^; the mean operative time was 47 minutes (SD, 15 minutes). A mean of 3 Opus Magnum (Smith & Nephew) anchors were used (range, 3-9 anchors) (Table 1).

**Table 1:** Participant Characteristics^*a*^

Characteristic	Value, n = 164
Men	108 (66)
Mean age at surgery, y	64 ± 9.7 (39-91)
Right shoulders	98 (60)
Primary surgery	101 (62)
Full-thickness tear	164 (100)
Mean anteroposterior tear size, mm	36 ± 11.3 (10-80)
Mean mediolateral tear size, mm	31 ± 10.7 (10-70)
Mean tear size area, cm^2^	12
Mean operative time, min	47 ± 14.9 (15-100)
Mean number of anchors^*b*^ used	3 (3-9)

a Values are presented as mean ± SD (range) or n (%).

b Opus Magnum anchors (Smith & Nephew).

### Patch Overview

One interposition PTFE patch (0.6%) was inserted by open surgery in 2000. Twenty interposition PTFE patches (12%) were inserted by an arthroscopic mattress method^18^ and 143 patches (87%) were inserted by an arthroscopic weave method.^20^ Two types of interposition PTFE patches were used during the study period: 1.85-mm Bard PTFE felt (C. R. Bard) (n = 146) and 2-mm Gore-Tex expanded PTFE (Gore Medical) (n = 18). There was no difference in functional failure between patch types (*P* = .06).

### Adverse Events

One patient experienced infection of the interposition PTFE patch at 1 month postoperatively and was managed with a course of oral antibiotics, irrigation, and patch removal. One patient (0.6%) experienced a foreign body reaction to the patch at 1 year postoperatively and had the patch removed. No other adverse events were identified.

### Patient-Reported Pain and Function

**Figure 4.**
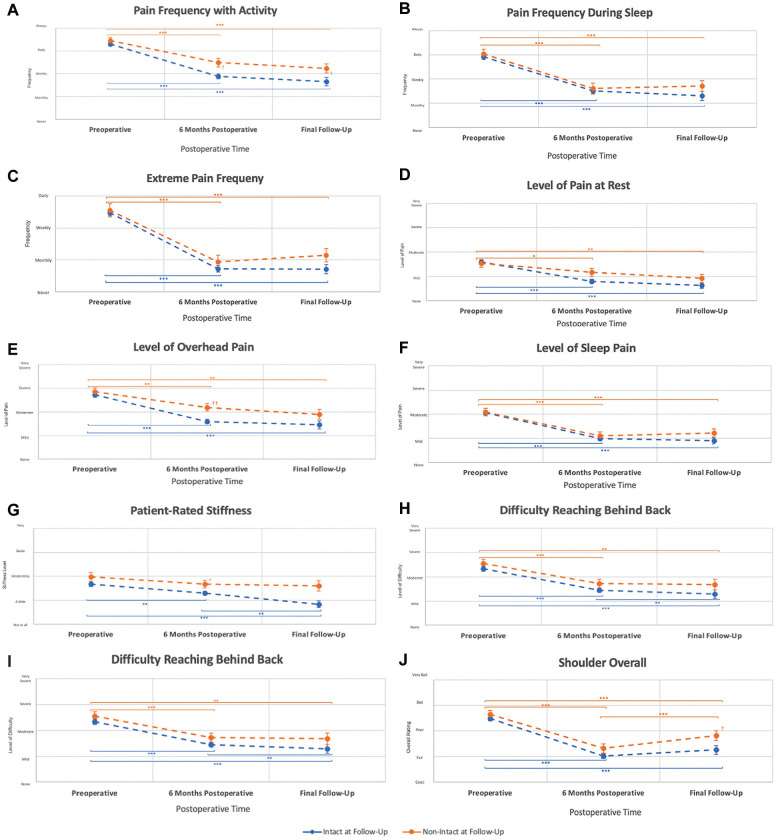
Patient-reported outcomes comparison between intact and nonintact interposition PTFE patch repairs. (A) Pain frequency with activity. (B) Pain frequency during sleep. (C) Extreme pain frequency. (D) Level of pain at rest. (E) Level of overhead pain. (F) Level of sleep pain. (G) Patient-reported stiffness. (H) Difficulty reaching overhead. (I) Difficulty reaching behind back. (J) Overall shoulder rating. Values represent the mean ± standard error of the mean. The follow-up had a median of 26 months. *P < .05, **P < .01, ***P < .001, Wilcoxon-signed rank test. ^†^*P* < .05, ^††^*P* < .01, ^†††^*P* < .001, Mann-Whitney *U* test. n = 164. PTFE, polytetrafluoroethylene.

At 6 months after interposition PTFE patch repair, all patients reported improved pain and function from preoperative levels (*P* < .05). This improvement was more pronounced at the final follow-up in patients with interposition PTFE patches, which were intact on ultrasound, compared with patients with nonintact interposition PTFE patches (Figure 4). Specifically, frequency of activity pain (*P* = .04), patient-reported stiffness (*P* = .008), level of difficulty reaching overhead (*P* = .01), and overall shoulder rating (*P* = .03) were greater in intact interposition PTFE patches at the final follow-up compared with nonintact interposition PTFE patches (Table 2).

**Table 2:** Comparing Patient-Reported Responses Between Patients With Intact and Nonintact Interposition PTFE Patches on Ultrasound at the Final Follow-up^*a*^

Shoulder Function at Final Follow-up	Mean Patient Response		Mann-Whitney *U* Test, *P*
	Intact	Nonintact	
Activity pain frequency	Monthly-weekly	Weekly-daily	.04^*b*^
Stiffness	None-mild	Mild-moderate	.004^*c*^
Overhead difficulty	Mild-moderate	Moderate-severe	.009^*c*^
Overall shoulder rating	Mild-moderate	Mild-moderate	.034^*b*^

a PTFE, polytetrafluoroethylene.

b *P* < .05.

c *P* < .01.

### Strength

**Figure 5.**
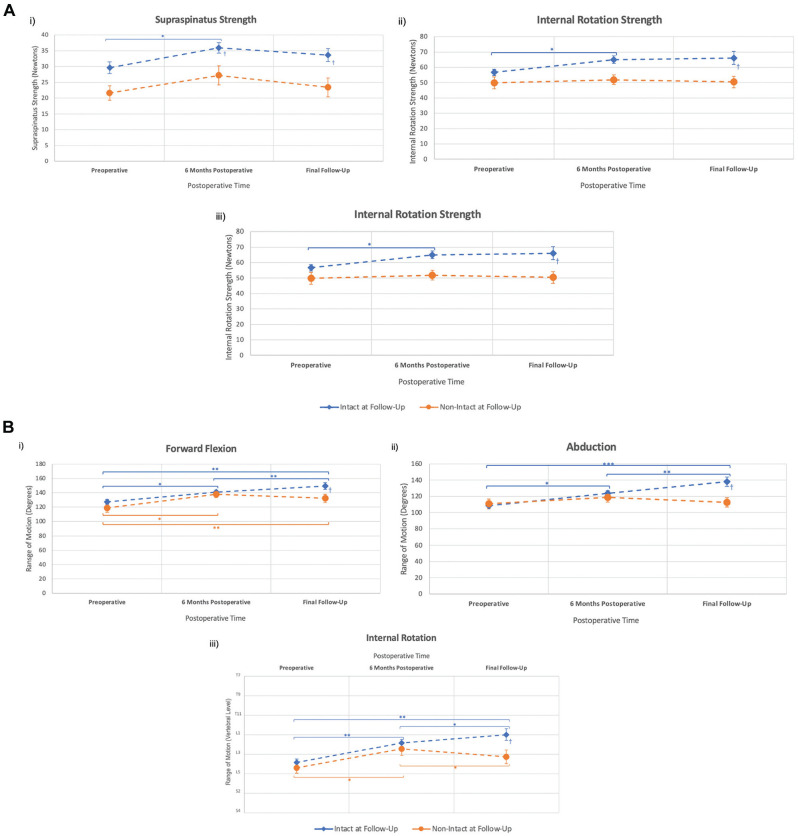
Objective outcomes after interposition PTFE patch repair. (A) Strength comparison between intact and nonintact PTFE patch repairs. Supraspinatus strength (*i*). Internal rotation strength (*ii*). External rotation strength (*iii*). (B) Range of motion comparison between intact and nonintact PTFE patch repairs. Forward flexion (*i*). Abduction (*ii*). Internal rotation (*iii*). Values represent mean ± standard error of the mean. The final follow-up had a median of 26 months. *P < .05, **P < .01, ***P < .001, Wilcoxon-Signed Rank test. ^†^*P* < .05, ^††^*P* < .01, ^†††^*P* < .001, Mann-Whitney *U* test. n = 164. PTFE, polytetrafluoroethylene.

Overall, patients demonstrated increased hand-held dynamometer strength by a mean of 5 N for internal rotation, 6 N for supraspinatus, and 6 N for external rotation at 6 months after interposition PTFE patch repair for massive and irreparable rotator cuff tears (*P* < .05). Patients with intact interposition PTFE patches on ultrasound had greater preoperative external rotation strength (*P* = .03) compared with patients with nonintact interposition PTFE patches. At the final follow-up, supraspinatus strength was 32% (11 N) greater (*P* = .02), external rotation was 39% (18 N) stronger (*P* = .001), and internal rotation was 23% (11 N) stronger (*P* = .04) in patients with intact interposition PTFE patches compared with patients with nonintact interposition PTFE patches (Figure 5A).

### Range of Motion

Overall, patients demonstrated an improved range of motion from preoperative levels by a mean of 16° for forward flexion, 12° for abduction, and 2 vertebral levels for internal rotation (*P* < .05) at 6 months postoperatively. At the final follow-up (median, 26 months), forward flexion was 12° greater (*P* = .045), abduction was 20° greater (*P* = .008), and internal rotation was 2 vertebral levels higher (*P* = .03) in patients with intact interposition PTFE patches compared with patients with nonintact interposition PTFE patches (Figure 5B).

### Integrity on Ultrasound

**Figure 6.**
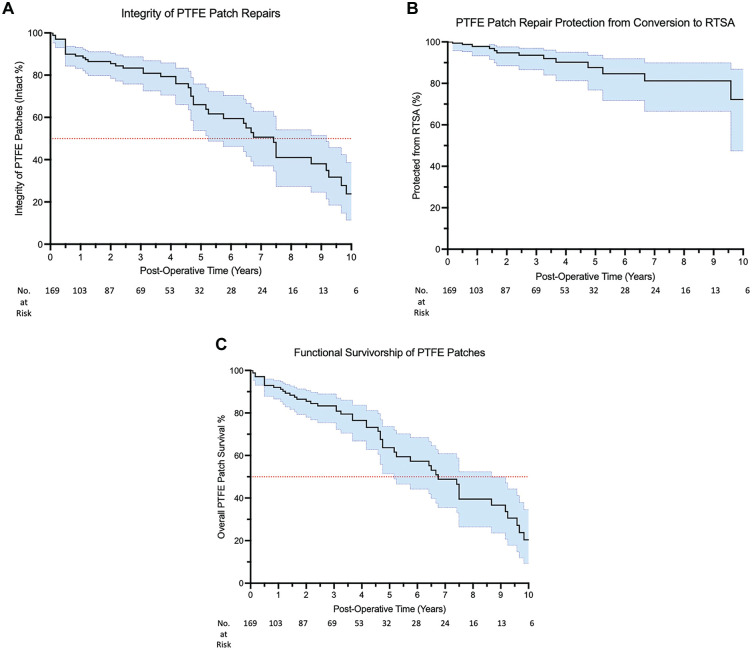
Kaplan-Meier Curves. (A) Kaplan-Meier survival curve for integrity of interposition PTFE patches. Loss of integrity is defined as the PTFE patch not being attached to the rotator cuff tendon or greater tuberosity on ultrasound. (B) Kaplan-Meier survival curve of participants progressing to reverse total shoulder arthroplasty after interposition PTFE patch repair. (C) Kaplan-Meier survival curve of functional survivorship of interposition PTFE patches. Functional failure is defined as the aggregate of participants whose interposition PTFE patch had lost integrity on ultrasound or who underwent removal of the interposition PTFE patch ± reverse total shoulder arthroplasty. 95% CIs are represented in blue. n = 169 (*including 5 PTFE patches, which failed before 6 months*). The red line represents 50% of intact PTFE patches. PTFE, polytetrafluoroethylene. RTSA, reverse total shoulder arthroplasty.

In the cohort of 164 patients with a minimum follow-up of 6 months, 42 (26%) interposition PTFE patches had lost integrity on ultrasound at their final follow-up (median, 26 months; range, 6 months to 18 years). Of those who had lost integrity on ultrasound, the median time to loss of integrity was 5 years (Figure 6A). of 164 interposition PTFE patches, 28 (17%) were nonintact at the patch-tendon interface, 4 (2%) had lost integrity at the bone-patch interface, and 8 (5%) were not attached at either interface. Two (1%) interposition PTFE patches were no longer visible on ultrasound at 8 and 10 years, respectively.

In the whole cohort, Kaplan-Meier analysis estimated that the median time until loss of integrity on ultrasound was 7.5 years (95% CI, 5.5-9.5 years).

### Conversion to Reverse Total Shoulder Arthroplasty

At the final follow-up (median, 26 months), 153 of 164 (93%) participants were free from conversion to reverse total shoulder arthroplasty after interposition PTFE patch repair for a massive and irreparable rotator cuff tear. Five of 42 (12%) participants with nonintact interposition PTFE patches on ultrasound went on to reverse total shoulder arthroplasty. In addition, 6 participants with intact interposition PTFE patches went on to reverse total shoulder arthroplasty (Figure 6B).

In the whole cohort, Kaplan-Meier analysis estimated that the median time for patient conversion to reverse total shoulder arthroplasty was 12 years after interposition PTFE patch insertion (95% CI, 8.5-15.6 years).

### Functional Failure

Functional failure of interposition PTFE patch repairs was defined as the aggregate of participants whose interposition PTFE patch had lost integrity on ultrasound or who underwent interposition PTFE patch removal with or without conversion to reverse total shoulder arthroplasty. Using this definition, 50 of 164 (30%) interposition PTFE patches had functionally failed at a median time of 5 years postoperatively (range, 6 months to 12 years) (Figure 6C). A total of 42 interposition PTFE patches functionally failed due to loss of integrity on ultrasound, and 8 PTFE patches failed due to removal with or without conversion to reverse total shoulder arthroplasty.

In the whole cohort, Kaplan-Meier analysis estimated that the median time for functional survivorship of interposition PTFE patch repairs was 7.4 years (95% CI, 6.5-8.3 years).

### Arthritis and Proximal Humeral Migration

**Figure 7.**
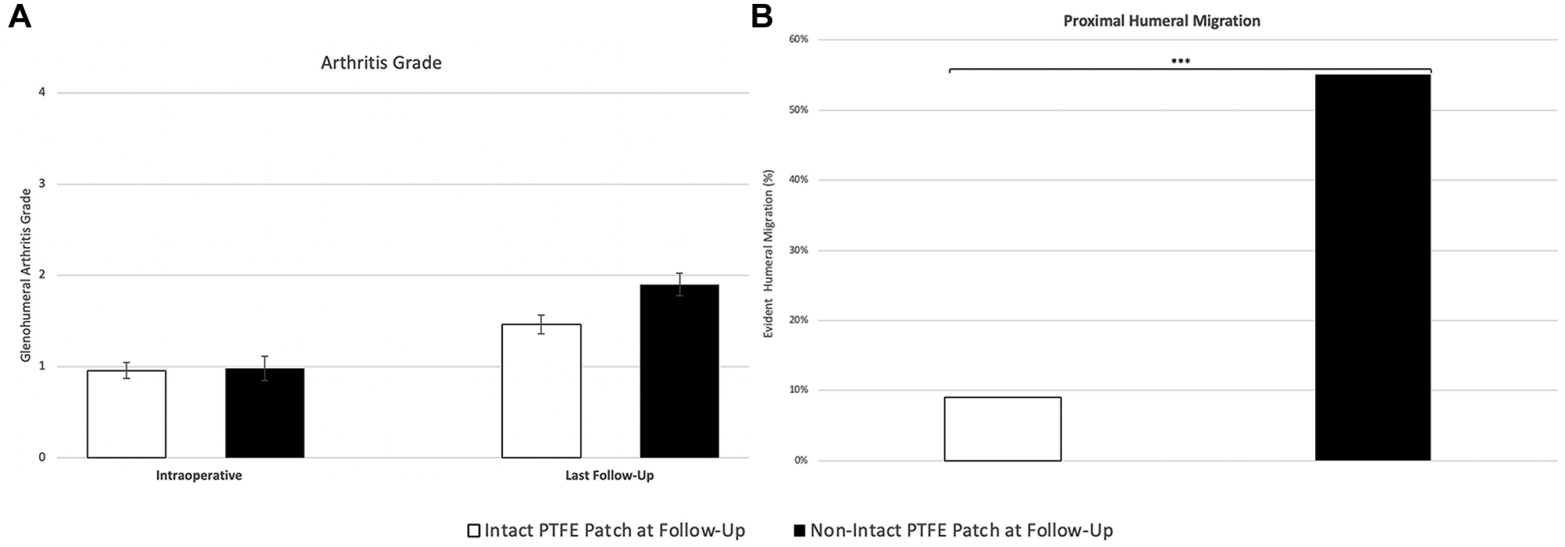
Glenohumeral arthritis and proximal humeral migration in shoulders with interposition PTFE patch repairs. (A) Glenohumeral arthritis, graded intra- and postoperatively at the final follow-up (median, 40 months; range, 6 months to 19 years). Values represent mean ± standard error of the mean. *P < .05, Mann-Whitney *U* test; Cohen's *d* effect size, 0.53; n = 110. (B) Proximal humeral migration was graded postoperatively at the final follow-up (median, 40 months; range, 6 months to 19 years). Values represent the total percentage of evident humeral migration in intact and nonintact PTFE patch groups. ***P < .001, chi-square analysis; Phi effect size = −0.5; n = 54. PTFE, polytetrafluoroethylene.

At the final follow-up appointment (median, 40 months; range, 6 months to 19 years), 110 of 164 (67%) participants underwent radiography. Patients with intact interposition PTFE patches on ultrasound had 21% less severe glenohumeral arthritis than patients with nonintact PTFE patches (*P* = .03; Cohen's *d* effect size, 0.53) (Figure 7A). A total of 54 of 164 (33%) participants had radiographs taken pre- and postoperatively for proximal humeral migration to be assessed. At the final follow-up (median, 40 months; range, 6 months to 19 years), patients with intact interposition PTFE patches were found to demonstrate 46% less proximal humeral migration than patients with nonintact interposition PTFE patches (*P* < .001, Phi effect size = −0.5) (Figure 7B).

## Discussion

The main findings of this study were that interposition PTFE patches inserted for massive and irreparable rotator cuff tears were safe and improved patient pain, function, shoulder strength, and range of motion from preoperative levels at 6 months. Moreover, these improvements persisted, especially if the patch remained intact on ultrasound. Of the interposition PTFE patches that functionally failed, the median survivorship time was 5 years. In the whole cohort, Kaplan-Meier analysis estimated that the median survivorship time of these PTFE patches was 7.4 years. At the final follow-up (median, 26 months), 93% of participants were free from conversion to reverse total shoulder arthroplasty. Patients with intact interposition PTFE patches had 21% less severe glenohumeral arthritis and demonstrated a 46% lower incidence of proximal humeral migration compared with patients with nonintact interposition PTFE patches.

Several interventions have been proposed for the management of massive and irreparable rotator cuff tears. A systematic review by Kovacevic et al^12^ compared physical therapy, debridement, partial repair, superior capsule reconstruction, balloon arthroplasty, reverse total shoulder arthroplasty, and interposition grafts. With a total of 37 articles and 2285 patients, the authors found that interposition graft repairs were the only intervention that resulted in improved patient-reported outcomes, measured by Constant-Murley and American Shoulder and Elbow Surgeon scores. Several graft types have been investigated for interposition repair, including autograft, allograft, extracellular, and synthetic options. Of these options, PTFE patches and polyethylene terephthalate have been found to have strong biomechanical properties and good construct integrity ex vivo.^1,7,14,15^ Our study furthers these biomechanical findings and demonstrates the clinical benefits of intact interposition PTFE patches.

Most studies on the clinical outcomes of interposition PTFE patch repairs for massive and irreparable rotator cuff tears have come from our institute. Previously, Seker et al^20^ had conducted the largest cohort study on interposition PTFE patches with a sample size of 58, and Sandhu et al^18^ had the most recent study with a sample size of 41. With an initial sample size of 164, our study stands as the largest investigation of interposition PTFE patch repairs for massive and irreparable rotator cuff tears from our institute and adds additional information to our previous work.^18,20,21^

While all patients reported improvements in pain and function from preoperative levels, patients with intact interposition PTFE patches on ultrasound reported less overhead pain and less difficulty reaching overhead at 6 months, correlating with less reported activity pain compared with patients with nonintact interposition PTFE patches at 6 months. At the final follow-up, patients with intact interposition PTFE patches noted less pain with activity and reported a better overall shoulder rating compared with patients with nonintact interposition PTFE patches.

Between 6 months and their final follow-up, patients with intact interposition PTFE patches reported decreased stiffness, improved difficulty reaching behind their backs, and demonstrated improved range of motion across the board. These findings indicate that patients with intact interposition PTFE patches may experience greater improvements in patient-reported outcomes as postoperative time progresses.

At their final follow-up, patients with intact interposition PTFE patches reported less stiffness and were found to have greater abduction and internal rotation range of motion compared with patients with nonintact interposition PTFE patches. Patients with intact interposition PTFE patch repairs also had a 21% lower severity of glenohumeral arthritis and a 46% lower incidence of proximal humeral migration, and this might explain why patients with intact interposition PTFE patches had better range of motion and reported less stiffness than patients with nonintact patches.

In the cohort of 164 patients, 42 (26%) interposition PTFE patches had lost integrity on ultrasound at a median time of 5 years. Similar to Sandhu et al,^18^ we found that most (67%) interposition PTFE patches lost integrity on ultrasound at the patch-tendon interface.

A strength of this study was the large initial sample size (n = 164). Interposition PTFE patch integrity was assessed by a single, experienced musculoskeletal sonographer who was blinded to participants’ clinical outcomes. Data were collected prospectively using standardized methods, and a single surgeon performed all operations.

Nevertheless, there were limitations to this study. Participant follow-up had a median time of 26 months, which ideally could be longer, and there was a significant proportion of participants who did not return for follow-ups beyond 6 months. The numbers at risk tables for Kaplan-Meier analysis dropped significantly beyond 8 years, resulting in larger confidence intervals beyond this timepoint, which may affect long-term interpretations. While there was a comparison group, there was no control group that had not received PTFE patches. Lastly, external validity was limited as a single surgeon performed the interposition PTFE patch repairs, and other surgeons may not be able to reproduce our results if a different technique or rehabilitation protocol is implemented.

## Conclusion

This study demonstrated that interposition PTFE patch repairs for massive and irreparable rotator cuff tears improved patient pain, function, shoulder strength, and range of motion, especially if the PTFE patch remained intact on ultrasound. Patients with intact interposition PTFE patches demonstrated less glenohumeral arthritis and proximal humeral migration. Of 164 interposition PTFE patches, 50 (30%) functionally failed at a median time of 5 years. In the whole cohort, Kaplan-Meier analysis estimated that the median survivorship time of these PTFE patches was 7.4 years. At the final follow-up, 93% of participants were free from conversion to reverse total shoulder arthroplasty.

## Figures and Tables

**Appendix Figure A.**
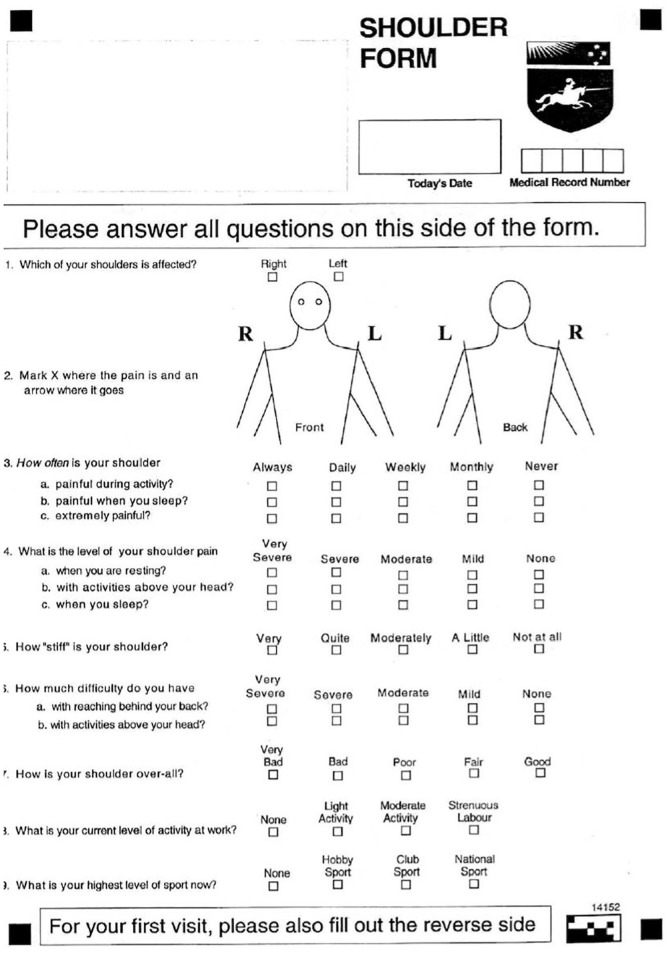
L’Insalata Shoulder Rating Questionnaire.
